# Prediction of Residual Stroke Risk in Anticoagulated Patients with Atrial Fibrillation: mCARS

**DOI:** 10.3390/jcm10153357

**Published:** 2021-07-29

**Authors:** Wern Yew Ding, José Miguel Rivera-Caravaca, Francisco Marin, Christian Torp-Pedersen, Vanessa Roldán, Gregory Y. H. Lip

**Affiliations:** 1 Liverpool Centre for Cardiovascular Science, University of Liverpool and Liverpool Heart & Chest Hospital, Liverpool L7 8TX, UK; dwyew@hotmail.com (W.Y.D.); jmrivera429@gmail.com (J.M.R.-C.); 2 Department of Cardiology, Hospital Clínico Universitario Virgen de la Arrixaca, University of Murcia, Instituto Murciano de Investigación Biosanitaria (IMIB-Arrixaca), CIBERCV, 30120 Murcia, Spain; fcomarino@hotmail.com; 3 Department of Cardiology and Clinical Research, Nordsjaellands Hospital, 3400 Hillerød, Denmark; christian.tobias.torp-pedersen@regionh.dk; 4 Department of Cardiology, Aalborg University Hospital, 9000 Aalborg, Denmark; 5 Department of Hematology and Clinical Oncology, Hospital General Universitario Morales Meseguer, University of Murcia, 30008 Murcia, Spain; vroldans@gmail.com; 6 Aalborg Thrombosis Research Unit, Department of Clinical Medicine, Aalborg University, 9000 Aalborg, Denmark

**Keywords:** atrial fibrillation, stroke risk, residual, anticoagulation, personalised, prediction tool

## Abstract

Our ability to evaluate residual stroke risk despite anticoagulation in atrial fibrillation (AF) is currently lacking. The Calculator of Absolute Stroke Risk (CARS) has been proposed to predict 1-year absolute stroke risk in non-anticoagulated patients. We aimed to determine whether a modified CARS (mCARS) may be used to assess the residual stroke risk in anticoagulated AF patients from ‘real-world’ and ‘clinical trial’ cohorts. We studied patient-level data of anticoagulated AF patients from the real-world Murcia AF Project and AMADEUS clinical trial. Individual mCARS were estimated for each patient. None of the patients were treated with non-vitamin K antagonist oral anticoagulants. The predicted residual stroke risk was compared to actual stroke risk. 3503 patients were included (2205 [62.9%] clinical trial and 1298 [37.1%] real-world). There was wide variation of CARS for each category of CHA_2_DS_2_-VASc score in both cohorts. Average predicted residual stroke risk by mCARS (1.8 ± 1.8%) was identical to actual stroke risk (1.8% [95% CI, 1.3–2.4]) in the clinical trial, and broadly similar in the real-world (2.1 ± 1.9% vs. 2.4% [95% CI, 1.6–3.4]). AUCs of mCARS for prediction of stroke events in the clinical trial and real-world were 0.678 (95% CI, 0.598–0.758) and 0.712 [95% CI, 0.618–0.805], respectively. mCARS was able to refine stroke risk estimation for each point of the CHA_2_DS_2_-VASc score in both cohorts. Personalised residual 1-year absolute stroke risk in anticoagulated AF patients may be estimated using mCARS, thereby allowing an assessment of the absolute risk reduction of treatment and facilitating a patient-centred approach in the management of AF. Such identification of patients with high residual stroke risk could help target more aggressive interventions and follow-up.

## 1. Introduction

Atrial fibrillation (AF) remains a major public health issue as it poses a significant risk of stroke and mortality [1,2,3]. There are several elements to the management of patients with AF, including regular and detailed risk assessments for stroke and bleeding [4]. These assessments are the cornerstone for enabling clinicians to provide appropriate anticoagulation-related recommendations [5,6]. In this regard, the benefit of treatment must outweigh any potential risk. Furthermore, the patient-centred nature of AF management dictates active communication of such information to patients during a shared-decision making process [7].

To this end, numerous risk models are available to predict the stroke risk in AF [8,9]. Nevertheless, each of these was designed for using in a non-anticoagulated AF population and intended to help classify patients in a dichotomous fashion (high vs. low risk) rather than provide values of absolute risk. Hence, there is currently no validated model to predict residual absolute stroke risk among anticoagulated patients with AF.

Recently, Lee et al. proposed the Calculator of Absolute Stroke Risk (CARS), which provides an estimation of the personalised 1-year absolute risk of stroke [10]. The authors studied this flexible risk-factor based model in a large ‘real-world’ Danish cohort of non-anticoagulated patients with first-diagnosed AF, and found some advantages over the widely used CHA_2_DS_2_-VASc score [8]. Although both models consist of similar clinical components, CARS deals with the supplied information differently in that age is included as a continuous variable and it takes into account the individual contribution of specific risk factors, thereby requiring an online calculator [10]. The use of CARS in a clinical trial cohort where stroke events are carefully adjudicated has not been previously described.

In this study, we aimed to determine whether a modified version of CARS may be used to assess the residual stroke risk in anticoagulated patients with AF in real-world (Murcia AF Project) and clinical trial (AMADEUS [Evaluating the Use of SR34006 Compared to Warfarin or Acenocoumarol in Patients with Atrial Fibrillation] trial) cohorts.

## 2. Materials and Methods

For the present analysis, we included patients from the Murcia AF Project and AMADEUS trial with a follow-up duration of 1-year or a stroke prior to this. The design of both studies have previously been described [11,12]. In brief, the Murcia AF Project was an observational study from a tertiary hospital in Spain that enrolled consecutive outpatients between May and December 2007 with non-valvular AF on stable vitamin K antagonist (VKA) therapy (i.e., International Normalised Ratio [INR] of 2.0 to 3.0) in the preceding six months. The initial period of stable INR minimised heterogeneity, thus avoiding confounding factors due to differences in the quality of anticoagulation control at study entry. The time in therapeutic range was re-calculated after six months. Patients with a rheumatic mitral or prosthetic heart valve, as well as those with any acute coronary syndrome, stroke, haemodynamic instability, and hospital admission or surgical intervention in the preceding six months were excluded.

The AMADEUS trial was a multicentre, randomised, open-label non-inferiority study with blinded adjudication of outcomes comparing fixed-dose idraparinux vs. dose-adjusted VKA in patients with non-valvular AF. Recruitment took place between September 2003 and July 2005. Patients with an indication for anticoagulation other than AF, transient AF caused by a reversible disorder, active bleeding or high-risk of bleeding, creatinine clearance of less than 10 mL/min, severe liver disease, uncontrolled hypertension, and recent or anticipated invasive procedures with potential for uncontrolled bleeding were excluded.

A complete medical history was recorded at inclusion and the recorded parameters were used to calculate the CHA_2_DS_2_-VASc score [8]. The 1-year absolute stroke risk without anticoagulation was determined using the online CARS (https://hjerteforeningen.shinyapps.io/riskvisrr/) [13]. Residual 1-year stroke risk with anticoagulation (‘mCARS’) was estimated using prior evidence of a 64% risk reduction in treated patients [14]. In this regard, mCARS was derived by multiplying the calculated CARS by 0.36.

In the Murcia AF Project, ischaemic stroke was defined as the sudden onset of a focal neurological deficit in a location consistent with the territory of a major cerebral artery due to an obstruction documented by imaging, surgery, or autopsy. All events in the AMADEUS trial were adjudicated by a central committee, who were blinded to treatment assignment. Events were limited to those that occurred within 1-year to enable a comparison between the risk of actual events and CARS, which was designed to estimate the 1-year absolute stroke risk.

The study protocol of the Murcia AF Project was performed in accordance with the ethical standards laid down in the 1964 Declaration of Helsinki and approved by the Ethics Committee from University Hospital Morales Meseguer. De-identified patient level data was used from the AMADEUS trial.

### Statistical Analyses

Continuous baseline variables were expressed using median and interquartile range (IQR), and tested for differences with Kruskal-Wallis test. Categorical variables were expressed using absolute frequencies and percentages, and tested for differences using chi-squared test.

Predicted 1-year stroke risks by CARS and CHA_2_DS_2_-VASc were compared using Wilcoxon signed rank test. Actual stroke risk at 1-year was determined as a percentage with 95% confidence intervals (CI). The predictive performance of mCARS for stroke events was investigated using receiver-operating characteristic (ROC) curves, and tested against the CHA_2_DS_2_-VASc score. Area under the curve (AUC) was used to reflect the c-index, which represents the ability of scores to predict events. A two-sided *p* value of <0.05 was considered statistically significant. Analyses were performed using SPSS software version 24.0 (SPSS, Inc., Chicago, IL, USA) and MedCalc v. 16.4.3 (MedCalc Software bvba, Ostend, Belgium).

## 3. Results

The study included 3503 patients with non-valvular AF: 2205 (62.9%) clinical trial and 1298 (37.1%) real-world patients with a stable INR 6 months prior to recruitment. Baseline demographics for the clinical trial cohort are summarised in Table S1. Median age was 71 (IQR 65–77), with 34.6% females. The prevalence of hypertension, diabetes mellitus, and prior thromboembolism were 75.9%, 19.5%, and 23.7%, respectively. Median CHA_2_DS_2_-VASc score was 3 (IQR 2–4) with a distribution of: 1 (*n* = 178; 8.1%), 2 (*n* = 463; 21.0%), 3 (*n* = 572; 25.9%), 4 (*n* = 486; 22.0%), and ≥5 (*n* = 506; 22.9%). None of the patients were treated with non-vitamin K antagonist oral anticoagulants.

Baseline demographics for the real-world cohort are summarised in Table S2. Median age was 76 (IQR 70–81), with 51.8% females. The prevalence of hypertension, diabetes mellitus, and prior thromboembolism were 81.7%, 26.1% and 4.1%, respectively. Median CHA_2_DS_2_-VASc score was 4 (IQR 3–5) with a distribution of: 0 (*n* = 17; 1.3%), 1 (*n* = 66; 5.1%), 2 (*n* = 138; 10.6%), 3 (*n* = 270; 20.8%), 4 (*n* = 336; 25.9%), and ≥5 (*n* = 471; 36.3%). In both cohorts, patients with increasing CHA_2_DS_2_-VASc score were significantly older (*p* < 0.001 each) with greater comorbidities including anaemia (*p* < 0.001 each).

### 3.1. CARS, mCARS, and Actual Stroke Risk Based on CHA_2_DS_2_-VASc Score

The CARS, mCARS, and actual stroke risk at 1-year according to CHA_2_DS_2_-VASc score was broadly similar across both the clinical trial and real-world cohorts (Table 1). Importantly, both CARS and mCARS increased with greater CHA_2_DS_2_-VASc score. Nonetheless, there was a wide variation of CARS with extreme outliers for each category of CHA_2_DS_2_-VASc score in both cohorts (Table 2).

### 3.2. Predicted 1-Year Stroke Risk by CARS vs. CHA_2_DS_2_-VASc

Predicted stroke risks by CARS and CHA_2_DS_2_-VASc were comparable in the clinical trial (median CARS 2.9% [IQR 2.0–5.2] vs. CHA_2_DS_2_-VASc 3.2% [IQR 2.2–4.8], *p* = 0.794) but not in the real-world (median CARS 3.8% [IQR 2.6–5.5] vs. CHA_2_DS_2_-VASc 4.8% [IQR 3.2–7.2], *p* = 0.002 (Table 3). The upper limit of predicted stroke risk by CARS was noticeably higher compared to CHA_2_DS_2_-VASc in both cohorts.

### 3.3. Predicted vs. Actual 1-Year Stroke Risk

At 1-year, there were 40 (1.8%) and 31 (2.4%) stroke events in the clinical trial and real-world cohorts, respectively. The average predicted residual stroke risk by mCARS (1.8 ± 1.8%) was identical to the actual risk of stroke events, despite anticoagulation (1.8% [95% CI 1.3–2.4]) in the clinical trial and broadly similar in the real-world cohort (2.1 [± 1.9%] by mCARS vs. 2.4% [95% CI 1.6–3.4]). Additionally, these values were comparable across the various subgroups stratified by the CHA_2_DS_2_-VASc score in both cohorts.

**Figure 1:**
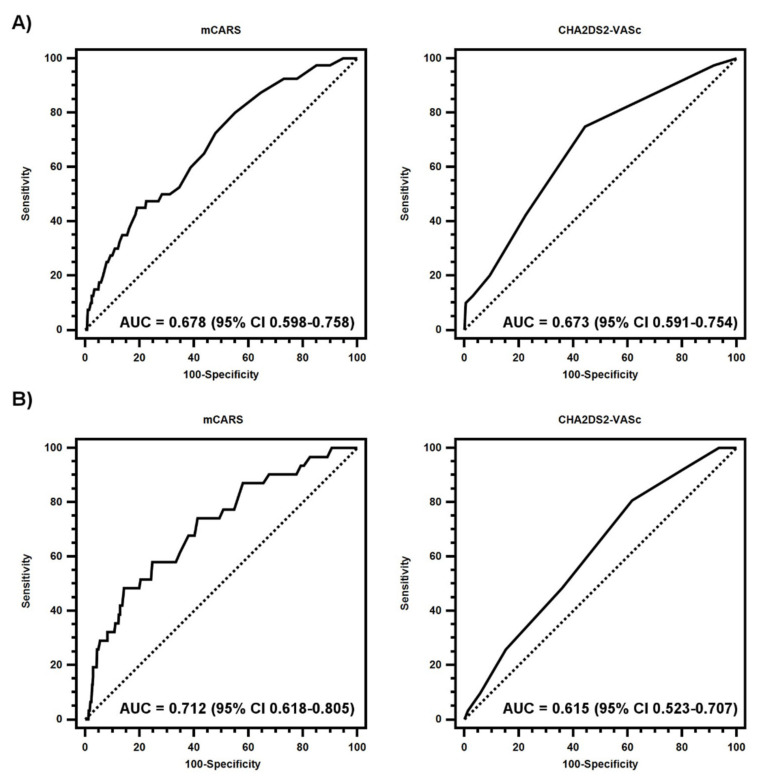
Receiver-operating characteristic curves for 1-year stroke events by mCARS and CHA_2_DS_2_-VASc score in the clinical trial (**A**) and real-world (**B**) cohorts.

Using ROC curve analyses, the AUC of mCARS and CHA_2_DS_2_-VASc score for prediction of stroke events in the clinical trial cohort were similar, 0.678 (95% CI 0.598–0.758) and 0.673 (95% CI 0.591–0.754), respectively (Figure 1). Thus, the performance of both these scores were not significantly different in the clinical trial cohort (*p* = 0.859).

In the real-world cohort, mCARS performed significantly better than the CHA_2_DS_2_-VASc score for prediction of stroke events (AUC 0.712 [95% CI 0.618–0.805] vs. AUC 0.615 [95% CI 0.523–0.707], *p* = 0.001).

### 3.4. Exploratory Analysis

**Figure 2:**
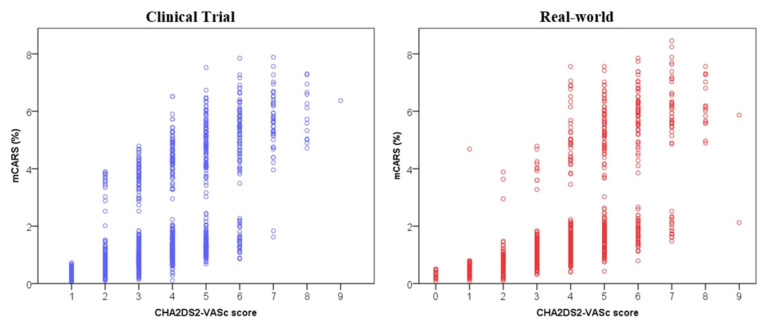
mCARS vs. CHA_2_DS_2_-VASc score in clinical trials and the real-world.

The distribution of mCARS based on the CHA_2_DS_2_-VASc score is shown in Figure 2. In an exploratory analysis, we found that mCARS was able to refine stroke risk estimation for each point of the CHA_2_DS_2_-VASc score in both clinical trial and real-world cohorts (Table 4).

## 4. Discussion

In this study of patients with AF, we demonstrated that: (1) there was considerable variation in the predicted stroke risk by CARS for each category of the CHA_2_DS_2_-VASc score; (2) the residual stroke risk determined by mCARS closely resembled the number of stroke events despite anticoagulation; and (3) mCARS was superior to the CHA_2_DS_2_-VASc score for predicting the residual stroke risk despite anticoagulation therapy, in the real-world cohort. However, the predictive performance of both these scores were not significantly different in the clinical trial cohort with adjudicated stroke events.

Our findings provide a means to quantify the absolute risk reduction offered by anticoagulation therapy among individuals with AF, in everyday clinical practice. In this regard, the absolute risk reduction may be derived from a simple comparison between CARS (pre-anticoagulation risk) and mCARS (post-anticoagulation risk) to provide an accurate marker for evaluating the benefit of specific treatments in AF. In daily practice, stroke risk is estimated using the CHA_2_DS_2_-VASc score and weighed against the estimated bleeding risk associated with anticoagulation therapy, in order to determine the optimal treatment for patients with AF. However, this approach assumes that anticoagulation will completely nullify any stroke risk, which is not true [15]. While this risk is reduced by anticoagulation, some patients may still remain at disproportionately high risk of residual stroke. These patients may be identified using mCARS for more aggressive interventions, such as catheter ablation [16,17] and adherence to the ABC pathway [18], and close follow-up.

Given the increasing treatment options and potential hazards associated with each [19,20,21,22], both CARS and mCARS may be useful to assist in the clinician-patient communication of stroke risk and the benefits of anticoagulation in AF. In this sense, a detailed discussion centred on absolute risk (as offered by CARS and mCARS) will be more readily appreciable by patients compared to relative risk, which has a tendency to exaggerate the perception of difference [23].

For research purposes, mCARS provides a reliable method for estimating residual stroke risk despite anticoagulation. This is particularly applicable to single-arm studies that focus on alternative methods of stroke prevention in AF [24], as it allows the stroke risk following intervention to be compared to estimated risk if anticoagulation therapy had been utilised instead. Furthermore, with increasing evidence of the benefits of catheter ablation in AF [25,26,27,28], this tool may be used to identify patients with a high residual stroke risk despite anticoagulation, who may benefit from more aggressive treatment (and follow-up). Importantly, we have shown that mCARS remains broadly valid in the context of both clinical trial and real-world cohorts, although in terms of stroke prediction, the performance of mCARS was similar to the CHA_2_DS_2_-VASc score, if based on the adjudicated outcomes of a clinical trial cohort. A possible explanation for this lack of difference may be related to the age of clinical trial participants, which were close to the cut-off thresholds of the CHA_2_DS_2_-VASc score, and therefore accounted for by this model. Alternatively, mCARS may perform better in the real-world, given that it was derived from CARS, which was designed using real-world data.

Overall the CHA_2_DS_2_-VASc score remains a valuable clinical asset, providing useful information on stroke risk in a simplified and practical manner. The strength of this approach lies in its ability to identify low-risk patients who may not derive net benefit from anticoagulation therapy [8]. However, the wide variability of individual stroke risk in each category of the CHA_2_DS_2_-VASc score, shown in this study, highlights the simple reductionist nature of this clinical score. Indeed, mCARS was able to refine stroke risk estimation for each point of the CHA_2_DS_2_-VASc score. Also, the components of the CHA_2_DS_2_-VASc score do not carry equal weight for stroke, and also change differentially over time [29,30]. The advantage of CARS is that it addresses some of these issues and emphasises the point that stroke risk in AF is a continuum. It may further add to the holistic and individualised management of patients with AF. Nevertheless, its calculation is complex and may not be suitable for busy clinical settings.

### Limitations

Given that the findings from this study were based on a post-hoc analysis of the AMADEUS trial and a single-centre Caucasian population in the Murcia AF Project, it should therefore be interpreted with caution and may not be representative of the wider cohort of AF patients. There were no patients who were treated with a non-vitamin K antagonist oral anticoagulant in this study. Nonetheless, the results remain pertinent as VKA therapy is still used widely, especially in certain population groups such as those with severe chronic kidney disease. Furthermore, as patients from the Murcia AF Project were required to have a stable INR prior to enrolment, the findings from this study may be more transferable to patients on non-vitamin K antagonist oral anticoagulant. As we censored the outcomes at one year, it remains unclear whether our results would be applicable to periods of extended follow-up. However, both the AMADEUS trial and Murcia AF Project enrolled patients with a variety of stroke risk factors, which is reflected by the wide distribution of CHA_2_DS_2_-VASc score, thereby strengthening the validity of mCARS as a useful tool in AF. In determining mCARS, the relative risk reduction of anticoagulation therapy was assumed to be constant across the whole cohort.

## 5. Conclusions

A personalised residual 1-year absolute risk of stroke in anticoagulated patients with AF may be estimated using mCARS. This allows an assessment of the absolute risk reduction of treatment and facilitates a patient-centred approach in the management of AF. Such identification of patients with high residual stroke risk could help target more aggressive interventions and follow-up.

## Figures and Tables

**Table 1:** CARS, mCARS, and actual stroke risk stratified by CHA_2_DS_2_-VASc score in clinical trials vs. the real-world.

	Clinical Trial			Real-World		
CHA_2_DS_2_-VASc	CARS (IQR)	mCARS (IQR)	Actual Stroke Risk (95% CI)	CARS (IQR)	mCARS (IQR)	Actual Stroke Risk (95% CI)
0	NA			0.9 (0.6–1.3)	0.3 (0.2–0.5)	0 (0–0)
1	1.1 (0.7–1.4)	0.4 (0.3–0.5)	0.6 (0–1.7)	1.4 (0.9–1.7)	0.5 (0.3–0.6)	0 (0–0)
2	2.0 (1.5–2.4)	0.7 (0.5–0.9)	0.9 (0–1.7)	2.1 (1.5–2.6)	0.8 (0.5–0.9)	1.4 (0.2–5.2)
3	2.6 (2.1–3.4)	0.9 (0.8–1.2)	0.9 (0.1–1.6)	2.8 (2.5–3.4)	1.0 (0.9–1.2)	1.5 (0.4–3.8)
4	3.6 (2.8–5.6)	1.3 (1.0–2.0)	2.7 (1.2–4.1)	3.9 (3.3–5.5)	1.4 (1.2–1.8)	3.0 (1.4–5.5)
5	6.7 (3.6–14.1)	2.4 (1.3–5.1)	3.1 (1.1–5.0)	4.8 (3.9–12.2)	1.7 (1.4–4.4)	2.6 (1.0–5.4)
6	13.6 (5.5–15.8)	4.9 (2.0–5.7)	2.2 (0–4.7)	12.8 (4.8–16.7)	4.6 (1.7–6.0)	4.0 (1.3–9.4)
7	15.7 (14.5–17.4)	5.7 (5.2–6.3)	1.7 (0–5.1)	15.6 (5.9–17.5)	5.6 (2.1–6.3)	3.4 (0.4–12.2)
8	16.5 (14.0–18.5)	5.9 (5.0–6.7)	28.6 (1.5–55.6)	16.9 (15.7–19.5)	6.1 (5.7–7.0)	5.9 (0.1–32.8)
9	17.7 (17.7–17.7)	6.4 (6.4–6.4)	0 (0–0)	11.1 (5.9–16.3)	4.0 (2.1–5.9)	0 (0–0)

CI, confidence interval; IQR, interquartile range; NA, not applicable.

**Table 2:** Range of absolute 1-year stroke risk stratified by CHA_2_DS_2_-VASc score in clinical trials vs. the real-world.

	Range of Absolute 1-Year Stroke Risk (%)	
	Clinical Trial	Real-World
CHA_2_DS_2_-VASc score 0	NA	0.2–1.4
CHA_2_DS_2_-VASc score 1	0.2–2.0	0.2–13.0
CHA_2_DS_2_-VASc score 2	0.3–10.8	0.3–10.8
CHA_2_DS_2_-VASc score 3	0.4–13.3	0.9–13.3
CHA_2_DS_2_-VASc score 4	0.3–18.1	1.1–21.0
CHA_2_DS_2_-VASc score 5	1.9–20.9	1.2–21.0
CHA_2_DS_2_-VASc score 6	2.4–21.8	2.2–21.8
CHA_2_DS_2_-VASc score 7	4.5–21.9	4.1–23.5
CHA_2_DS_2_-VASc score 8	13.1–20.3	13.6–21.0

NA, not applicable.

**Table 3:** Comparison of overall predicted 1-year stroke risk by CARS vs. CHA_2_DS_2_-VASc.

	Clinical Trial		Real-World	
	% Risk	*p* value	% Risk	*p* value
CHA_2_DS_2_-VASc predicted stroke risk				
Mean (SD)	4.3 (2.6)		5.3 (2.8)	
Median (IQR)	3.2 (2.2–4.8)		4.8 (3.2–7.2)	
Range	0.6–12.2		0.2–12.2	
CARS predicted stroke risk				
Mean (SD)	5.1 (4.9)		5.7 (5.2)	
Median (IQR)	2.9 (2.0–5.2)		3.8 (2.6–5.5)	
Range	0.2–21.9	0.794*	0.2–23.5	0.002*

IQR, interquartile range; SD, standard deviation. * CARS vs. CHA_2_DS_2_-VASc predicted stroke risk.

**Table 4:** Breakdown of actual stroke risk based on mCARS for subgroups of the CHA_2_DS_2_-VASc score.

	Clinical Trial			Real-World		
	**Number of Patients**	**Events, n**	**Actual Stroke Risk (95% CI), %**	**Number of Patients**	**Events, n**	**Actual Stroke Risk (95% CI), %**
CHA_2_DS_2_-VASc score 0	NA			17	0	0 (0–0)
mCARS <1%				17	0	0 (0–0)
mCARS 1–2%				0	0	NA
mCARS 2–5%				0	0	NA
mCARS >5%				0	0	NA
CHA_2_DS_2_-VASc score 1	178	1	0.6 (0–1.7)	66	0	0 (0–0)
mCARS <1%	178	1	0.6 (0–1.7)	65	0	0 (0–0)
mCARS 1–2%	0	0	NA	0	0	NA
mCARS 2–5%	0	0	NA	1	0	0 (0–0)
mCARS >5%	0	0	NA	0	0	NA
CHA_2_DS_2_-VASc score 2	463	4	0.9 (0–1.7)	138	2	1.4 (0.2–5.2)
mCARS <1%	389	4	1.0 (0–2.0)	121	2	1.7 (0.2–6.0)
mCARS 1–2%	54	0	0 (0–0)	14	0	0 (0–0)
mCARS 2–5%	20	0	0 (0–0)	3	0	0 (0–0)
mCARS >5%	0	0	NA	0	0	NA
CHA_2_DS_2_-VASc score 3	572	5	0.9 (0.1–1.6)	270	4	1.5 (0.4–3.8)
mCARS <1%	303	2	0.7 (0–1.6)	107	0	0 (0–0)
mCARS 1–2%	200	2	1.0 (0–2.4)	153	4	2.6 (0.7–6.7)
mCARS 2–5%	69	1	1.4 (0–4.3)	10	0	0 (0–0)
mCARS >5%	0	0	NA	0	0	NA
CHA_2_DS_2_-VASc score 4	486	13	2.7 (1.2–4.1)	336	10	3.0 (1.4–5.5)
mCARS <1%	91	1	1.1 (0–3.3)	44	1	2.3 (0.06–12.7)
mCARS 1–2%	275	6	2.2 (0.4–3.9)	229	7	3.1 (1.2–6.3)
mCARS 2–5%	101	4	4.0 (0.1–7.8)	48	2	4.2 (0.5–15.0)
mCARS >5%	19	2	10.5 (0–25.7)	15	0	0 (0–0)
CHA_2_DS_2_-VASc score 5	295	9	3.1 (1.1–5.0)	269	7	2.6 (1.0–5.4)
mCARS <1%	16	0	0 (0–0)	16	0	0 (0–0)
mCARS 1–2%	128	4	3.1 (0.1–6.2)	153	1	0.7 (0.01–3.6)
mCARS 2–5%	76	3	3.9 (0–8.4)	45	1	2.2 (0.06–12.4)
mCARS >5%	75	2	2.7 (0–6.4)	55	5	9.1 (3.0–21.2)
CHA_2_DS_2_-VASc score 6	137	3	2.2 (0–4.7)	124	5	4.0 (1.3–9.4)
mCARS <1%	3	0	0 (0–0)	1	0	0 (0–0)
mCARS 1–2%	34	1	2.9 (0–8.9)	42	0	0 (0–0)
mCARS 2–5%	37	0	0 (0–0)	25	0	0 (0–0)
mCARS >5%	63	2	3.2 (0–7.6)	56	5	8.9 (2.9–20.8)
CHA_2_DS_2_-VASc score 7	59	1	1.7 (0–5.1)	59	2	3.4 (0.4–12.2)
mCARS <1%	0	0	NA	0	0	NA
mCARS 1–2%	2	0	0 (0–0)	13	0	0 (0–0)
mCARS 2–5%	10	0	0 (0–0)	9	0	0 (0–0)
mCARS >5%	47	1	2.1 (0–6.4)	37	2	5.4 (0.7–19.5)
CHA_2_DS_2_-VASc score 8	14	4	28.6 (1.5–55.6)	17	1	5.9 (0.1–32.8)
mCARS <1%	0	0	NA	0	0	NA
mCARS 1–2%	0	0	NA	0	0	NA
mCARS 2–5%	4	0	0 (0–0)	2	0	0 (0–0)
mCARS >5%	10	4	40.0 (3.1–76.9)	15	1	6.7 (0.2–37.1)
CHA_2_DS_2_-VASc score 9	1	0	0 (0–0)	2	0	0 (0–0)
mCARS <1%	0	0	NA	0	0	NA
mCARS 1–2%	0	0	NA	0	0	NA
mCARS 2–5%	0	0	NA	1	0	0 (0–0)
mCARS >5%	1	0	0 (0–0)	1	0	0 (0–0)

CI, confidence interval; NA, not applicable.

## Data Availability

The data that support the findings of this study are available from the corresponding author, G.Y.H.L., upon reasonable request.

## Supplementary Materials

The following are available online at https://www.mdpi.com/article/10.3390/jcm10153357/s1, Table S1: Baseline characteristics stratified by the CHA_2_DS_2_-VASc score in clinical trials, Table S2: Baseline characteristics stratified by the CHA_2_DS_2_-VASc score in the real-world.
