# Supplementary material for: Prediction of Residual Stroke Risk in Anticoagulated Patients with Atrial Fibrillation: mCARS
Source: J Clin Med. 2021 Jul 29;10(15):3357. doi: 10.3390/jcm10153357 (PMC8348193; doi:10.3390/jcm10153357)
Supplement: Supplementary file 1 [file jcm-10-03357-s001.zip › jcm-1265938-supplementary.pdf]

# Supplementary Materials

# Prediction of Residual Stroke Risk in Anticoagulated Patients with Atrial Fibrillation: mCARS

**Wern Yew Ding [1,†], José Miguel Rivera-Caravaca [1,2,†], Francisco Marin [2], Christian Torp-Pedersen [3,4], Vanessa Roldán [5,‡] and Gregory Y. H. Lip [1,6,‡,*]**

[1] Liverpool Centre for Cardiovascular Science, University of Liverpool and Liverpool Heart & Chest Hospital, Liverpool, UK; dwyew@hotmail.com (W.Y.D.); jmrivera429@gmail.com (J.M.R.-C.)
[2] Department of Cardiology, Hospital Clínico Universitario Virgen de la Arrixaca, University of Murcia, Instituto Murciano de Investigación Biosanitaria (IMIB-Arrixaca), CIBERCV, 30120 Murcia, Spain; fcomarino@hotmail.com
[3] Department of Cardiology and Clinical Research, Nordsjaellands Hospital, 3400 Hillerød, Denmark; christian.tobias.torp-pedersen@regionh.dk
[4] Department of Cardiology, Aalborg University Hospital, 9000 Aalborg, Denmark
[5] Department of Hematology and Clinical Oncology, Hospital General Universitario Morales Meseguer, University of Murcia, 30008 Murcia, Spain; vroldans@gmail.com
[6] Aalborg Thrombosis Research Unit, Department of Clinical Medicine, Aalborg University, 9000 Aalborg, Denmark
* Correspondence: gregory.lip@liverpool.ac.uk
† These authors contributed equally to this work.
‡ These authors contributed equally to this work.

## Supplementary Tables

**Table S1**. Baseline characteristics stratified by $CHA_2DS_2$-VASc score in Clinical Trial

| $CHA_2DS_2$-VASc score | Total (n = 2205) | 1 (n = 178) | 2 (n = 463) | 3 (n = 572) | 4 (n = 486) | ≥5 (n = 506) | *p* value |
|---|---|---|---|---|---|---|---|
| Age (years), median (IQR) | 71 (65 - 77) | 58 (53 - 62) | 68 (61 - 72) | 71 (66 - 75) | 75 (69 - 78) | 76 (72 - 80) | <0.001 |
| Age groups (years), n (%) | | | | | | | <0.001 |
| 18 - 39 | 3 (0.1%) | 2 (1.1%) | 1 (0.2%) | 0 (0%) | 0 (0%) | 0 (0%) | |
| 40 - 54 | 107 (4.9%) | 49 (27.5%) | 22 (4.8%) | 23 (4.0%) | 10 (2.1%) | 3 (0.6%) | |
| 55 - 64 | 423 (19.2%) | 123 (69.1%) | 156 (33.7%) | 90 (15.7%) | 33 (6.8%) | 21 (4.2%) | |
| 65 - 74 | 892 (40.5%) | 4 (2.2%) | 227 (49.0%) | 304 (53.1%) | 196 (40.3%) | 161 (31.8%) | |
| >75 | 780 (35.4%) | 0 (0%) | 57 (12.3%) | 155 (27.1%) | 247 (50.8%) | 321 (63.4%) | |
| Female sex, n (%) | 762 (34.6%) | 0 (0%) | 58 (12.5%) | 192 (33.6%) | 221 (45.5%) | 291 (57.5%) | <0.001 |
| eGFR, median (IQR) | 87 (70 - 94) | 67 (55 - 85) | 84 (66 - 92) | 87 (70 - 94) | 87 (75 - 94) | 90 (81 - 97) | <0.001 |
| Comorbidities, n (%) | | | | | | | |
| Anaemia | 135 (11.3%) | 3 (3.1%) | 17 (6.9%) | 32 (10.7%) | 29 (11.3%) | 54 (18.2%) | <0.001 |
| Coronary artery disease | 699 (31.7%) | 3 (1.7%) | 71 (15.3%) | 155 (27.1%) | 179 (36.8%) | 291 (57.5%) | <0.001 |
| Diabetes mellitus | 429 (19.5%) | 0 (0%) | 40 (8.6%) | 93 (16.3%) | 116 (23.9%) | 180 (35.6%) | <0.001 |
| Heart failure | 536 (24.3%) | 29 (16.3%) | 71 (15.3%) | 131 (22.9%) | 112 (23.0%) | 193 (38.1%) | <0.001 |
| Hypertension | 1673 (75.9%) | 142 (79.8%) | 303 (65.4%) | 393 (68.7%) | 390 (80.2%) | 445 (87.9%) | <0.001 |
| Prior thromboembolism | 522 (23.7%) | 0 (0%) | 21 (4.5%) | 69 (12.1%) | 118 (24.3%) | 314 (62.1%) | <0.001 |
| Prior stroke or TIA | 470 (21.3%) | 0 (0%) | 20 (4.3%) | 64 (11.2%) | 108 (22.2%) | 278 (54.9%) | <0.001 |
| Vascular disease | 699 (31.7%) | 3 (1.7%) | 71 (15.3%) | 155 (27.1%) | 179 (36.8%) | 291 (57.5%) | <0.001 |

eGFR, estimated glomerular filtration rate; IQR, interquartile range; TIA, transient ischaemic attack.

**Table S2**. Baseline characteristics stratified by $CHA_2DS_2$-VASc score in Real-World

| $CHA_2DS_2$-VASc score | Total (n = 1298) | 0 (n = 17) | 1 (n = 66) | 2 (n = 138) | 3 (n = 270) | 4 (n = 336) | ≥5 (n = 471) | *p* value |
|---|---|---|---|---|---|---|---|---|
| Age (years), median (IQR) | 76 (70 - 81) | 58 (53 - 63) | 63 (56 - 68) | 69 (61 - 73) | 73 (69 - 78) | 78 (73 - 81) | 79 (76 - 83) | <0.001 |
| Age groups (years), n (%) | | | | | | | | <0.001 |
| 18 - 39 | 0 (0.0%) | 0 (0.0%) | 0 (0.0%) | 0 (0.0%) | 0 (0.0%) | 0 (0.0%) | 0 (0.0%) | |
| 40 - 54 | 32 (2.5%) | 6 (35.3%) | 11 (16.7%) | 11 (8.0%) | 1 (0.4%) | 2 (0.6%) | 1 (0.2%) | |
| 55 - 64 | 128 (9.9%) | 11 (64.7%) | 31 (47.0%) | 38 (27.5%) | 28 (10.4%) | 12 (3.6%) | 8 (1.7%) | |
| 65 - 74 | 380 (29.3%) | 0 (0.0%) | 24 (36.4%) | 73 (52.9%) | 127 (47.0%) | 86 (26.6%) | 70 (14.9%) | |
| >75 | 758 (58.4%) | 0 (0.0%) | 0 (0%) | 16 (11.6%) | 114 (42.2%) | 236 (70.2%) | 392 (83.2%) | |
| Female sex, n (%) | 672 (51.8%) | 0 (0.0%) | 7 (10.6%) | 27 (19.6%) | 108 (40.0%) | 198 (58.9%) | 332 (70.5%) | <0.001 |
| eGFR, median (IQR) | 71 (59 - 86) | 84 (69 - 90) | 81 (66 - 93) | 75 (62 - 88) | 72 (61 - 87) | 71 (57 - 85) | 68 (54 - 83) | <0.001 |
| Comorbidities, n (%) | | | | | | | | |
| Anaemia | 234 (18.0%) | 1 (5.9%) | 6 (9.1%) | 15 (10.9%) | 28 (10.4%) | 74 (22.0%) | 110 (23.4%) | <0.001 |
| Coronary artery disease | 238 (18.3%) | 0 (0.0%) | 3 (4.5%) | 17 (12.3%) | 33 (12.2%) | 49 (14.6%) | 136 (28.9%) | <0.001 |
| Diabetes mellitus | 339 (26.1%) | 0 (0.0%) | 3 (4.5%) | 14 (10.1%) | 35 (13.0%) | 85 (25.3%) | 202 (42.9%) | <0.001 |
| Heart failure | 399 (30.7%) | 0 (0.0%) | 4 (6.1%) | 17 (12.3%) | 39 (14.4%) | 86 (25.6%) | 253 (53.7%) | <0.001 |
| Hypertension | 1061 (81.7%) | 0 (0.0%) | 26 (39.4%) | 90 (65.2%) | 220 (81.5%) | 293 (87.2%) | 432 (91.7%) | <0.001 |
| Prior thromboembolism | 53 (4.1%) | 0 (0.0%) | 1 (1.5%) | 0 (0.0%) | 2 (0.7%) | 9 (2.7%) | 41 (8.7%) | <0.001 |
| Prior stroke or TIA | 240 (18.5%) | 0 (0.0%) | 0 (0%) | 3 (2.2%) | 9 (3.3%) | 37 (11.0%) | 191 (40.6%) | <0.001 |
| Vascular disease | 284 (21.9%) | 0 (0.0%) | 3 (4.5%) | 17 (12.3%) | 36 (13.3%) | 54 (16.1%) | 174 (36.9%) | <0.001 |

eGFR, estimated glomerular filtration rate; IQR, interquartile range; TIA, transient ischaemic attack
